# AE&M's first issue of the year: important news!

**DOI:** 10.20945/2359-3997000000109

**Published:** 2019-02-01

**Affiliations:** 1 Universidade de São Paulo Universidade de São Paulo Faculdade de Medicina Hospital das Clínicas São Paulo SP Brasil Editor-chefe da Archives of Endocrinology and Metabolism, chefe da Unidade de Neuroendocrinologia, Serviço de Endocrinologia e Metabologia, Hospital das Clínicas, Faculdade de Medicina da Universidade de São Paulo (HCFMUSP), São Paulo, SP, Brasil

Dear readers, the *Archives of Endocrinology and Metabolism* is entering its fourth year of activities (1), and this issue brings three important news which I would like to share with you:

- First, the end of the Journal´s printed version, following the general trend to just keep the online version. This approach will make the AE&M more dynamic and agile.
- Second, the disclosure of our current impact index: 2.380. This achievement probably reflects the increase of international reporting of our Journal, with the consequent increase of the number of submissions and citations.
- Third, due to many commitments, Dr. Rui Maciel is leaving as an Associate Editor of the thyroid area, being replaced by Dr. Ana Luiza Maia. Additionally, Dr. Beatriz Schaan will reinforce the team of diabetes/obesity. I heartily thank Dr. Maciel for his important contribution to the AE&M and gladly welcome Drs. Maia and Schaan.

As Editor-in-Chief, I would like to acknowledge and thank the devoted and essential collaboration of our Associate Editors and Reviewers, as well as the Brazilian Endocrine Society (SBEM) Board of Directors for their constant support. Last but not least, I also want to extend my special thanks to the editorial team of the AE&M.
